# DockEM: an enhanced method for atomic-scale protein–ligand docking refinement leveraging low-to-medium resolution cryo-EM density maps

**DOI:** 10.1093/bib/bbaf091

**Published:** 2025-03-10

**Authors:** Jing Zou, Wenyi Zhang, Jun Hu, Xiaogen Zhou, Biao Zhang

**Affiliations:** College of Information Engineering, Zhejiang University of Technology, 288 Liuhe Road, Liuxia Street, Xihu District, Hangzhou 310023, China; Westlake AI Therapeutics Lab, Westlake Laboratory of Life Sciences and Biomedicine, Hangzhou 310024, China; Chinese Academy of Medical Sciences Suzhou Institute of Systems Medicine, Suzhou 215123, China; College of Information Engineering, Zhejiang University of Technology, 288 Liuhe Road, Liuxia Street, Xihu District, Hangzhou 310023, China; College of Information Engineering, Zhejiang University of Technology and Chinese Academy of Medical Sciences, Suzhou Institute of Systems Medicine, Suzhou 215123, China

**Keywords:** docking, cryo-EM, protein–ligand, REMC simulation, refinement

## Abstract

Protein–ligand docking plays a pivotal role in virtual drug screening, and recent advancements in cryo-electron microscopy (cryo-EM) technology have significantly accelerated the progress of structure-based drug discovery. However, the majority of cryo-EM density maps are of medium to low resolution (3–10 Å), which presents challenges in effectively integrating cryo-EM data into molecular docking workflows. In this study, we present an updated protein–ligand docking method, DockEM, which leverages local cryo-EM density maps and physical energy refinement to precisely dock ligands into specific protein binding sites. Tested on a dataset of 121 protein–ligand compound, our results demonstrate that DockEM outperforms other advanced docking methods. The strength of DockEM lies in its ability to incorporate cryo-EM density map information, effectively leveraging the structural information of ligands embedded within these maps. This advancement enhances the use of cryo-EM density maps in virtual drug screening, offering a more reliable framework for drug discovery.

## Introduction

Bioinformatics is a crucial field to study big data [1–4]. It plays an important role to deciphering high-resolution protein structures, drug development, and protein targeting which involved in progression of different diseases, including various types of cancers [5–7]. Many vital biological functions within organisms depend on the interactions between proteins and other intracellular molecules [8–10]. Molecular docking is a theoretical method used to study the interactions between proteins and ligands. One of the key applications of molecular docking is in the development of innovative drugs. However, new drug development is a costly and inefficient process, even with modern automated high-throughput screening technologies, developing a typical small-molecule drug can take several years and cost millions of dollars [11]. Molecular docking can rapidly and efficiently screen millions of compound molecules computationally [12]. Enhancing docking accuracy is crucial for the success of virtual drug screening and its application within the drug discovery pipeline [13].

Advances in cryo-electron microscopy (cryo-EM) technology have significantly increased its relevance in structure-based drug discovery [14]. Improvements in cryo-EM resolution have enabled the exploration of more potential drug targets, including membrane proteins, within structure-based drug discovery, expanding our understanding of drug target identification, design, and development, particularly in areas where conventional techniques, such as X-ray crystallography, face limitations [15, 16]. While cryo-EM density maps can achieve atomic-level resolution [17], the majority of maps stored in the Electron Microscopy Data Bank (EMDB) [18] are still deposited at medium to low resolutions (above 3 Å). Therefore, integrating effective medium to low-resolution cryo-EM density map data into the molecular docking process remains a pressing challenge that needs to be addressed.

Given the significance of docking, several methods have been specifically developed for protein–peptide docking, such as MOTR [19] and DGMOEA [20]. MOTR is an innovative multi-objective metaheuristic algorithm designed for the protein–peptide docking problem, while DGMOEA is a dual-population multi-objective evolutionary algorithm. Our work focuses primarily on the docking problem between proteins and small molecules, which differ substantially from peptides in terms of flexibility, size, and binding mechanisms. Currently, numerous molecular docking computational methods have been developed for small molecules, including ChemEM [14], AutoDock Vina [21], DOCK6 [22], EDock [23], CB-Dock2 [24], and EMERALD [25]. However, traditional molecular docking methods, such as AutoDock Vina and EDock, have certain fundamental limitations. These methods rely on conformation search strategies, guided by various composite energy functions, to quantify the interactions between ligand-protein compound during the molecular docking process. Designing energy functions that can accurately and efficiently describe these interactions is a daunting task. CB-Dock2, an improved version of CB-Dock [26], introduces template-based search, allowing for the specification of protein binding sites and utilizing a new curvature-based cavity detection method calculating the center and size of the cavity before docking with the popular AutoDock Vina program. ChemEM proposes a new ChemDock scoring function and utilizes differential density maps, which extract the ligand portion from the entire density map. EMERALD leverages the docking capabilities of RosettaGenFF and GA optimization, while simultaneously integrating cryo-EM density data with the physically realistic force field of RosettaGenFF. However, both methods have a limitation: the resolution of the density maps is typically low, and the resolution near the binding site may be compromised due to ligand-induced perturbations. As a result, the differential density maps and pseudo-atomic skeleton (derived from the unmodeled density map around the ligand) obtained under these conditions may lack some essential information. Despite the successes of these docking methods, achieving a balance between accuracy and efficiency in current molecular docking methods remains a challenge under certain conditions. Recently, methods specifically designed for fitting small molecules into cryo-EM density maps have emerged [25, 27]. The success of these processes depends on the initial fitting of the ligand, as positioning the ligand close to the correct site, combined with structural information from the density map, increases the chances of generating high-quality docking conformations.

**Figure 1:**
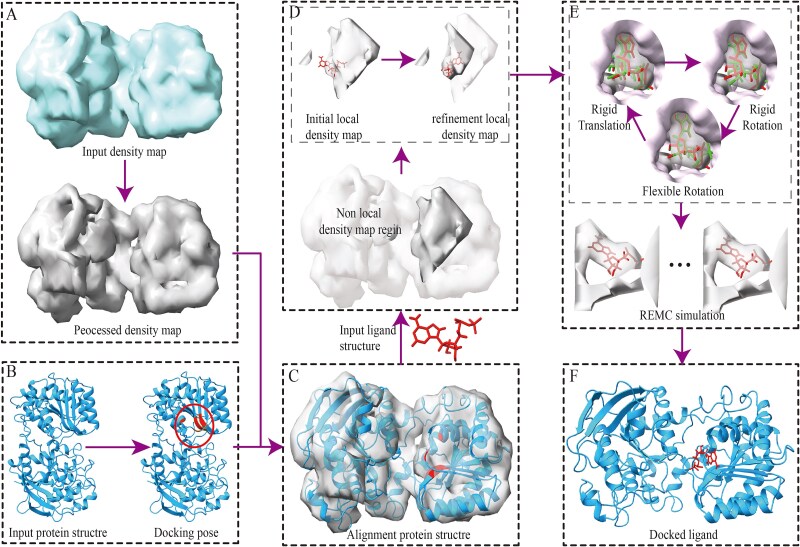
Workflow of protein–ligand docking using DockEM. (A) Input cryo-EM density map and processing. (B) Input structures of the protein and ligand, along with binding site information (or docking position) and cryo-EM density map. (C) Superpose the input protein structure onto the processed density map. (D) Use binding site information to locate the initial region of the local density map and update the center position of this initial local density map. (E) Perform conformational sampling through REMC simulation. (F) Select the final docked ligand structure.

In this work, we introduce DockEM, an updated molecular docking method designed for high-quality ligand position. DockEM achieves high-precision docking results using medium to low-resolution density maps. Starting from the three-dimensional structure of the protein, such as the receptor protein model generated by the structure prediction algorithm AlphaFold2 (AF2) [28] used in this study, DockEM identifies the approximate position of the ligand using binding site information through a local structure comparison method and extracts an initial local density map, as illustrated in Fig. 1A–C. The ligand structure is subsequently fitted to the initial local density map, with continuous optimization of the ligand’s position and the center position of the local ligand density map, as shown in Fig. 1D. A replica exchange Monte Carlo (REMC) [29] simulation, guided by our designed energy function that incorporates cryo-EM density map data, is employed to perform rigid-body alignment of the ligand with the local density map as well as flexible docking of the ligand to the protein, as illustrated in Fig. 1E and F. Extensive conformational searches and structural refinements are performed throughout the docking process. To rigorously assess the strengths and weaknesses of DockEM, we performed benchmark tests on 121 protein–ligand targets collected from DUDE [30] and COACH [31]. The standalone DockEM predictor used in this study is freely available at https://github.com/Hsfssfwa/DockEM.

## Materials and methods

### Dataset

The dataset used in our benchmark testing consists of 121 targets derived from the DUDE and COACH datasets referenced in EDock. After acquiring the protein–ligand datasets, we generated the corresponding simulated cryo-EM density map data using the EMAN2 [32] and UCSF Chimera [33] software packages. The resolutions of all simulated cryo-EM density maps ranged from 3 to 15 Å. The resolution range was chosen as it reflects typical medium to low-resolution scenarios [34] even though the average resolution of cryo-EM density maps in the community has been steadily improving over the past few years [35]. Details regarding the resolutions of all simulated density maps can be found in Supplementary File S1.

### Energy field

The force field in DockEM comprises a linear combination of the following four energy terms: the density map energy term, the ligand-protein intramolecular energy contributions from van der Waals interactions and Coulombic electrostatic interactions, the ligand’s internal van der Waals interactions, and the distance constraints to the local density map.


(1) \begin{equation*} {E}_{total}={w}_1{E}_{CC}+{w}_2{E}_{intra}+{w}_3{E}_{inter}+{w}_4{E}_{dis} \end{equation*}

Here, ${w}_1$, ${w}_2$, ${w}_3$ and ${w}_4$ represents the weight of different energy terms, where ${E}_{CC}=1- CC$. The term $CC$ refers to the correlation coefficient (CC) between the ligand structure and the local density map. The formula for calculating $CC$ is as follows:


(2) \begin{equation*} CC=\frac{\sum_{g=1}^{N_{vol}}\left[{\rho}_c(g)-\overline{\rho_c}\right]\left[{\rho}_e(g)-\overline{\rho_e}\right]}{\sqrt{\sum_{g=1}^N{\left[{\rho}_c(g)-\overline{\rho_c}\right]}^2}\sqrt{\sum_{g=1}^N{\left[{\rho}_e(g)-\overline{\rho_e}\right]}^2}} \end{equation*}

Here, $\overline{\rho_c}$ represents the average density value of the map inferred from the ligand structure, while $\overline{\rho_e}$ denotes the average density value of the experimental density map. ${N}_{vol}$ is the total number of grid points in the local density map. The term ${\rho}_e(g)$ refers to the density value at the *g*th grid point in the experimental density map, and ${\rho}_c(g)$ is the density value at the *g*th grid point in the density map inferred from the ligand structure. The value of ${\rho}_c(g)$ can be calculated as follows:


(3) \begin{equation*} {\rho}_c(g)={\sum}_{i=1}^{N_a}m\sqrt[2]{{\left(\frac{\pi^2}{{\left(2.4+0.8R\right)}^2}\right)}^3}\mathit{\exp}\left(-{\left(\frac{\pi }{2.4+0.8R}\right)}^2{\left|{x}_i-{x}_g\right|}^2\right) \end{equation*}

Here, ${N}_a$ represents the total number of atoms in the ligand structure, *m* is the atomic mass, $R$ denotes the resolution of the experimental density map, ${x}_i$ refers to the grid coordinates of the *i*th atom, and ${x}_g$ indicates the coordinates of the *g*th grid point.

The second term in Equation (1) accounts for the van der Waals and Coulombic electrostatic interactions between the ligand and the receptor:


(4) \begin{equation*} {E}_{intra}=\sum_{i\in protein}\sum_{j\in ligand}\left[{w}_{21}\left(\frac{A_{ij}}{d_{ij}^{12}}-\frac{B_{ij}}{d_{ij}^6}\right)+{w}_{22}\frac{q_i{q}_j}{4{d}_{ij}}\right] \end{equation*}

Here, ${w}_{21}$ and ${w}_{22}$ are the weights of this energy term, and ${d}_{ij}$ represents the distance between the *i*th atom in the protein and the *j*th atom in the ligand. Here, the protein atoms refer to those within the protein whose distance to the density map is less than half of the maximum distance between any two atoms within the ligand. ${A}_{ij}=\varepsilon{R}^{12}$ and ${B}_{ij}=2\varepsilon{R}^6$ are the repulsion and attraction parameters, respectively, where $R={r}_i+{r}_j$ and $\varepsilon =\sqrt{\varepsilon_i{\varepsilon}_i}$ are associated with the van der Waals radius and well depth, with values taken from the AMBER99 force field [36]. For the Coulomb interaction term, ${q}_i$ and ${q}_j$ are the charges of the atoms, determined using the Antechamber module of AMBER [37].

The third term in Equation (1) represents the van der Waals interactions within the ligand:


(5) \begin{equation*} {E}_{inter}=\sum_{i,j\in ligand,i\ne j}\left(\frac{A_{ij}}{s_{ij}^{12}}-\frac{B_{ij}}{s_{ij}^6}\right) \end{equation*}

Here, ${s}_{ij}$ is the distance between the *i*th atom and the *j*th atom within the ligand. In the second energy term, we calculated the van der Waals forces between the protein and the ligand, which effectively prevent conflicts between the protein and ligand atoms. Here, we calculate the van der Waals forces within the ligand to prevent internal conflicts between the ligand’s atoms.

The fourth term in Equation (1) represents the distance constraints to the local density map:


(6) \begin{equation*} {E}_{dis}=\left\{\begin{array}{@{}r}0,\kern0.5em \left|{c}_i-{c}_0\right|<{c}_{lim}\\{}\left|{c}_i-{c}_0\right|,\kern0.5em \left|{c}_i-{c}_0\right|\ge{c}_{lim}\end{array}\right. \end{equation*}

Here, ${c}_i$ is the center position of the conformation, ${c}_0$ is the center position of the local density map, and ${c}_{lim}$ is half of the maximum distance between any two atoms in the ligand. The purpose of setting this energy term is to reduce the confirmation search space of the ligand, allowing it to move around the ligand-specific region of the local density map to find a local optimum.

### Rigid-body alignment and local density map positioning

The protein is aligned with the global density map using REMC simulations guided by the ${E}_{CC}$ energy term (see *Energy field* section). Here, ${E}_{CC}$ represents the CC calculated between the protein structure and the cryo-EM density map. Starting from the ligand-protein binding site (or docking position), we define the region of the local ligand density map. The center of the binding site serves as the initial center of the local density map. If a docking position is provided, it is used as the center of the initial local density map. The local density map represented as a cubic region within the entire density map with an edge length twice the maximum distance between any two atoms in the ligand. The ligand is aligned to the binding site center and fitted to the local density map by maximizing the CC between the ligand and this map, while continuously optimizing the map’s center position. This fitting is achieved over 500 steps of Monte Carlo (MC) [38] simulation, guided by the energy function ${w}_1{E}_{CC}+{w}_2{E}_{intra}$ (see Equation (1)). After the simulation, the lowest-energy ligand conformation is selected, and the center of this ligand structure is set as the new center of the local density map. Based on this updated center, a cubic region with an edge length equal to the maximum ligand atom-to-atom distance is generated. The segment of the density map within this region becomes the local density map for further docking refinement.

### Ligand sampling by REMC

DockEM implements the REMC protocol, which enhances sampling efficiency and expands the sampling space. In this step, the REMC simulation includes $N$ replicas, which are sampled in parallel. The temperature of the *i*th replica is given by:


(7) \begin{equation*} {T}_i={T}_{min}{\left(\frac{T_{max}}{T_{min}}\right)}^{\frac{i-1}{N-1}} \end{equation*}

Here, ${T}_{min}=0.01$ represents the minimum temperature, and ${T}_{max}=1$ represents the maximum temperature. Every local MC step moves, a global exchange move is attempted between two neighboring replicas *i* and *j,* with the acceptance probability given by:


(8) \begin{equation*} {P}_{global}=\min \left\{1,\mathit{\exp}\left(\left({E}_j-{E}_i\right)\left(\frac{1}{K{T}_j}-\frac{1}{\mathrm{K}{T}_i}\right)\right)\right\} \end{equation*}

Here, ${E}_j$ and ${E}_i$ represent the energies of the *j*th and *i*th replicas, respectively, while ${T}_j$ and ${T}_i$ are the temperatures of the *j*th and *i*th replicas, respectively. $K$ is a parameter. This movement between neighboring replicas allows the conformation of a low-temperature replica to be exchanged with that of a high-temperature replica, thereby helping the low-temperature simulation escape from local energy minima.

### Refinement by flexible ligand-protein docking

Before performing flexible docking, we conducted an initial rigid protein–ligand docking, utilizing REMC simulations with 20 replicas. Each replica starts by positioning the ligand to the center of the local density map, with its initial state generated through random large-scale rotations and small-scale translations. Every 500 local MC moves, a global exchange move is attempted between two neighboring replicas. Following the REMC-based rigid protein–ligand docking, we selected the lowest-energy conformation from each of the 20 replicas to proceed flexible docking for further docking refinement. The flexible docking process is also implemented through REMC simulation, guided by the total energy ${E}_{total}$ (see Equation (1)). For flexible movements, single rotatable bonds are identified based on the “BOND” table in the MOL2 file. After rigid-body translation and rotation of the entire molecule, a random rotatable bond is chosen as the axis of rotation. All atoms distal to this bond are rotated by a random angle within the range of [−180°, 180°]. No translation is applied to these atoms during the process, as translating only a portion of the atoms could result in unnatural bond lengths and angles.

### Docking experiment evaluation indicators

Our docking performance is primarily assessed by the root mean square deviation (RMSD) of the docked ligand conformation relative to the native structure. Accurate RMSD calculation requires that the atom order in both the predicted and experimental ligand structures be consistent. However, we found cases where the atom order of some input ligand files differed from that of the experimental ligand files. This mismatch can result in discrepancies between the docked and experimental ligands, potentially leading to incorrect RMSD values. To address this issue, we employed DockRMSD [39] to standardize the atom order between the docked and experimental ligand structures before computing the RMSD.

## Results and discussion

### The overall docking results

To validate the DockEM method, comparisons were made with ChemEM and EMERALD, which similarly constrains ligand-protein docking using cryo-EM density; EDock, designed specifically for predicted models; and CB-Dock2, which employs templates to guide the docking pose. For ChemEM, the centroid of the binding site was specified as the location for ligand fitting, utilizing the center of the local density map identified by our method as the input centroid. For EMERALD, the ligand structure obtained after rigid docking by our method is used as the input ligand structure for EMERALD, while the protein structure fitted with the density map obtained from our method is used as the input protein structure for EMERALD. For EDock and CB-Dock2, additional binding site information was provided to ensure a fair comparison. In EDock, graph-based predicted binding site information was replaced with manually input data. CB-Dock2, an online server, allows users to input binding site information upon submission; if no information is provided, it defaults to blind docking. These steps ensured that the docking outcomes were based on consistent input data, allowing for an unbiased comparison among the five methods.

The protein structures in our dataset were predicted using AF2, encompassing 121 targets with an average TM-score of 0.983, as detailed in Supplementary File S2. These results indicate that AF2 can reliably produce correctly folded models for most protein receptors. It also demonstrates that for a given binding site (or docking position), the docking center calculated from the predicted protein structure is close to the true center, which facilitates subsequent iterative optimization and docking site searches. Table 1 summarizes the docking results. Overall, DockEM outperformed EMERALD, ChemEM, CB-Dock2, and EDock, achieving an average RMSD of 1.87 Å across the dataset. This represents a 10.1% reduction compared to EMERALD (2.06 Å), a 50.1% reduction compared to ChemEM (3.75 Å), a 35.1% reduction compared to CB-Dock2 (2.88 Å), and a 53.5% reduction compared to EDock (4.02 Å). Paired Student’s *t*-tests revealed statistically significant differences in ligand structures predicted by DockEM compared to those from EMERALD, ChemEM, CB-Dock2, and EDock, with *P*-values of 1.21E-2 for EMERALD, 1.24E-6 for CB-Dock2, 1.95E-23 for ChemEM, and 2.27E-23 for EDock.

**Table 1:** Summary of whole-protein docking results with known ligand binding sites

Method	DockEM	EMERALD	CB-Dock2	ChemEM	EDock
RMSD(Å)	1.87	2.06	2.88	3.75	3.99
Center distance(Å)	0.94	1.09	1.85	1.71	2.69
RMSD < 3 Å	110	95	71	40	45
Success rat	90.9%	78.5%	58.7%	33.1%	37.2%
*P*-value	–	1.21E−2	1.24E−6	1.95E−23	2.27E−23

The RMSD and Center Distance values represent the averages for all docked ligands in the dataset. Center distance represents the distance between the ligand center obtained using different methods and the native ligand center. The success rate is defined as the proportion of docked ligand structures exhibiting an RMSD of <3 Å in comparison to the native ligand structure.

**Figure 2:**
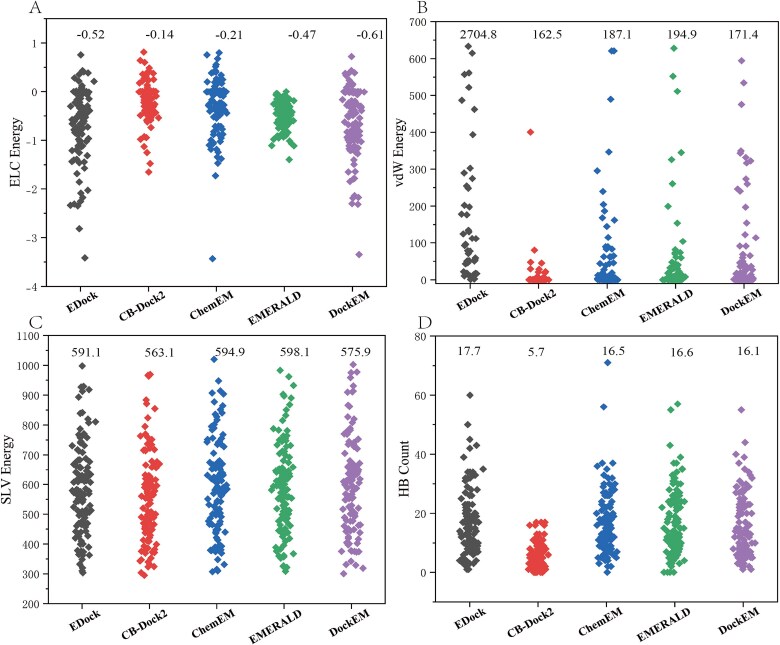
Comparison of five docking methods in four types of energies. All four energies are calculated between the protein and ligand structures. (A) Comparison of five docking methods in the electrostatic (ELC) energy term. (B) Comparison of five docking methods in the van der Waals (vdW) energy term. (C) Comparison of five docking methods in the solvation (SLV) energy term. (D) Comparison of five docking methods in the HB count. The numbers labeled in the figure represent the average value of each energy term.

To provide a more comprehensive comparison of the five docking methods, we used RDkit [40] to calculate the electrostatic (ELC), van der Waals (vdW), hydrogen bond (HB), and solvation (SLV) energies for the structures generated by each method. These energy-based metrics were used to further assess the protein–ligand interactions captured by the docking poses. The analysis highlights the comparative strengths of our method against four other methods (EDock, ChemEM, EMERALD, and CB-Dock2). As shown in Fig. 2A, in the ELC energy term, our method (−0.61) consistently ranked first, with only a marginal difference compared to EDock (−0.52), outperforming EMERALD (−0.47), CB-Dock2 (−0.14) and ChemEM (−0.21). For the vdW and SLV energy terms, the differences among the five methods were relatively small; however, our method ranked second in both cases, as shown in Fig. 2B and C. Regarding the HB energy term, RDkit counts the number of HBs formed between the protein and ligand. The HB count cannot be directly used to assess the quality of the HB energy. While a higher count generally indicates stronger and more stable interactions between the ligand and protein, an excessively high count may also suggest potential clashes between them. This is well illustrated in EDock, as shown in Fig. 2D, where the HB count of 17.7 is the highest among the five methods and EDock also exhibits the largest vdW energy of 2704.8. In contrast, CB-Dock2 has the smallest HB count of 5.7, with the lowest vdW energy of 162.5. for EMERALD, the HB count is 16.6 and the vdW energy is 194.9. Our method, with an HB count of 16.1 and a vdW energy of 171.4, demonstrates a balance between the HB and vdW energy terms. These energy results indicate that our method is competitive with the other approaches, as it not only produces docking poses with lower RMSD but also achieves relatively favorable energy values across the four physical energy terms. This dual advantage underscores the effectiveness and robustness of our method in generating high-quality protein–ligand docking models.

### Performance of alignment based on local ligand density map

**Figure 3:**
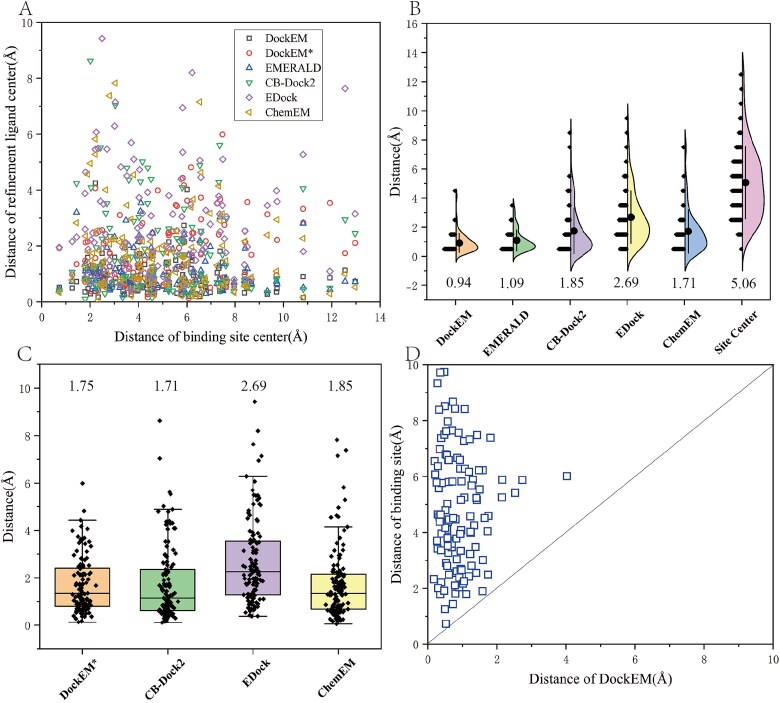
The statistic result of ligand and binding site in different methods. Here, DockEM and DockEM* refers to finally refinement ligand structure (flexible result) and the lowest-energy ligand structure (rigid docking result), respectively, in the DockEM method. (A) The relationship overview of distance of binding site center and distance of refinement ligand center for all methods. The *x*-axis represents distance of binding site center with native ligand center. The *y*-axis represents distance of refinement ligand center with native ligand center. All methods represented by different markers. (B) The statistic result of the distance between the refinement ligand center (or binding site center) and native ligand center for DockEM, EMERALD, CB-Dock2, EDock, ChemEM, and binding site. (C) The scatter plot of the distance between the ligand center of rigid docking and native ligand center obtained by the four methods: DockEM*, CB-Dock2, ChemEM, and EDock. (D) Comparison of the distances between the ligand center obtained by DockEM and the center of the binding site. The *x*-axis represents the distance between the DockEM-docked ligand center and the native ligand center; the *y*-axis represents the distance between the binding site center and the native ligand center.

In molecular docking, accurately positioning the ligand within the docking site is crucial for obtaining high-quality ligand structures in subsequent docking steps. DockEM extracts the local ligand density map after aligning the protein to the global density map using rigid-body superposition. This approach leverages the local density map and rigid-body alignment to precisely determine the docking position of the ligand on the protein. To assess the effectiveness of refining the ligand docking position using the local density map, we compared the distance of predicted binding site center and the distance of aligned ligand center with the distance of the ligand’s native structure center, as shown in the Fig. 3A and B.

**Figure 4:**
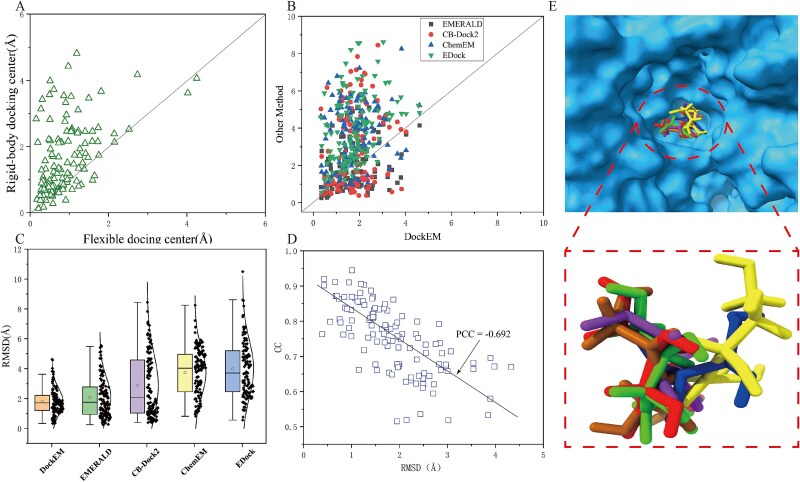
Results for docked ligand using 121 protein–ligand targets. (A) Comparison of the distances between the ligand centers after rigid docking and flexible docking. The *x*-axis represents the distance between the ligand center and the native center after flexible docking, while the y-axis represents the distance between the ligand center and the native center after rigid docking. (B) Scatter plot comparison of ligand RMSD values obtained by five methods. The *x*-axis represents the ligand RMSD values obtained by the DockEM method, while the y-axis shows the RMSD values obtained by EMERALD, CB-Dock2, ChemEM, and EDock. (C) Box plot comparison of ligand structure RMSD values obtained by five methods: DockEM, EMERALD, CB-Dock2, ChemEM, and EDock. The y-axis represents RMSD values. (D) Schematic representation of ligand docking for the 3II3A_BS03_XUL complex based on the AF2 predicted model. The RMSD values of the ligand structures obtained by DockEM, EMERALD, ChemEM, CB-Dock2, and EDock relative to the native ligand structure are 0.36 Å, 2.76 Å, 1.65 Å, 2.48 Å, and 3.05 Å, respectively. The background shows the predicted protein model with a TM-score of 0.99. (E) RMSD values of protein–ligand complexes versus CC values from the local density map in the dataset. The RMSD values on the x-axis and CC values on the y-axis. The solid line shows the linear regression fit for all data points, with a Pearson correlation coefficient (PCC) of −0.692.

The average distance between the ligand’s center after rigid docking and the native structure’s center was 1.75 Å, reflecting a 65.4% reduction (from an initial 5.06 Å) when compared to the average distance between the predicted binding site’s center and the native structure’s center. This significant discrepancy between the distance of predicted binding site center and the distance of native structure’s center can be attributed to limitations in binding site prediction accuracy and variations in ligand size and shape. When binding site predictions are accurate, smaller ligand molecules generally have their native structure centers closer to the predicted site center. However, even with accurate predictions, larger ligands may still have centers that deviate from the binding site center due to their size and complexity. We also compared the distance of ligand structures center which obtained using three other methods-ChemEM, CB-Dock2, and EDock-with the distance of native structure’s center. As shown in Table 1 and Fig. 3C, the average distance reductions were modest at −2.33% (ChemEM: 1.71 Å), 5% (CB-Dock2: 1.85 Å), and 34.9% (EDock: 2.69 Å). The reason EMERALD is not compared here is that the ligand structure after rigid docking was directly input into EMERALD. As shown in Fig. 3D and Fig. 4A, after completing flexible docking, our method demonstrated further performance improvements over other methods on this metric. The average distance between the ligand center and the native structure center was 0.94 Å. Compared to binding site, the center distance with the native structure center was reduced by 81.4%; compared to EMERALD, by 15.96%; compared to ChemEM, by 44.25%; compared to CB-Dock2, by 49.19%; and compared to EDock, by 65.06%. These results demonstrate that the rigid fitting component of our method is effective in accurately locating the ligand docking position, even before flexible docking is applied. This initial docking position advantage partly explains why our method achieves superior RMSD performance in the flexible docking stage. Detailed information on distances of the binding site center, the ligand’s center after rigid docking, the ligand’s center after flexible docking, and the native structure’s center for all dataset examples can be found in Supplementary File S3.

In a dataset of 121 protein–ligand complexes, DockEM successfully docked 110 ligands, yielding a success rate of 90.9%. Since the protein structures used in our study were predicted rather than experimentally determined, we relaxed the success criteria, defining successful docking as a ligand RMSD of <3 Å. DockEM demonstrated a substantially higher docking success rate than the other four methods, with improvements of 12.4% over EMERALD (success rate of 78.5%), 57.8% over ChemEM (success rate of 33.1%), 32.2% over CB-Dock2 (success rate of 58.7%), and 53.7% over EDock (success rate of 37.2%). Fig. 4B presents a scatter plot of ligand RMSD values obtained by DockEM, EMEALD, ChemEM, CB-Dock2, and EDock. Most blue triangles and green inverted triangle, representing ChemEM and EDock, are positioned above the diagonal line, indicating that DockEM significantly outperforms these methods. The black squares and red circles, representing EMERALD and CB-Dock2, exhibit improved performance in some instances, likely due to CB-Dock2’s ability to identify suitable templates for those specific cases and the pseudo-atomic skeleton constructed by EMERALD from the unmodeled density around the ligand provides valuable information., which leads to enhanced ligand docking structures. However, in the absence of a suitable template and lower-resolution density map, Ligand structures from CB-Dock2 and EMERALD deviate further from the native structure. In contrast, DockEM, which integrates density map information, achieves favorable results even in the absence of templates and when using lower-resolution density maps. As shown in Fig. 4C, under the strict criterion of RMSD <2 Å, DockEM identified 77 examples meeting this standard, compared to only 65 cases for EMERALD and 59 cases for CB-Dock2. Although EMERALD and ChemEM uses density map information to refine docking poses, its effectiveness is dependent on the resolution of the density maps. At lower resolutions (>6 Å), capturing ligand details accurately becomes challenging, leading to the loss of crucial ligand density data during the differencing process.

We present an example of a protein–ligand complex (PDB ID: 3II3A_BS03_XUL), shown in Fig. 4D. The predicted receptor protein achieved a TM-score of 0.99, with a density map resolution of 3.20 Å for both the protein and ligand. By incorporating density map data in the docking process, our DockEM method achieved a low RMSD of 0.36 Å for the docked ligand (illustrated in green in the figure), outperforming EMERALD (2.76 Å, brown), ChemEM (1.65 Å, purple), CB-Dock2 (2.48 Å, blue), and EDock (3.05 Å, yellow). A major factor in DockEM’s superior performance is its use of accurate density map information, which provides structural pose data not accessible to the other methods, with the partial exception of EMERALD and ChemEM. While the quality of this pose information relies on the map’s resolution, DockEM can effectively use position and orientation data from the density map by precisely identifying the region corresponding to the ligand referred to in our approach as the local density map. This allows DockEM to achieve significantly better docking accuracy and ligand refinement.

### Evaluating constructed models using CC

We calculated the CC value for the refined ligand conformations, considering only the ligand region within the local density map. We also tested other CC calculation methods but found these methods weren’t sensitive to conformation changes and worse than current local CC. A detailed comparison of the different CC calculation methods can be found in Supplementary File S4. To verify the effectiveness of the CC value in assessing the rigid docking between the ligand structure and the local density map, we analysis the CC values and RMSD values for all protein–ligand complexes along with their corresponding local density maps in the dataset, as shown in Fig. 4E. Here, the CC value is calculated specifically between the ligand and the corresponding ligand region within the density map. We calculated the Pearson CC between the CC value and the RMSD, finding a moderate negative correlation of −0.692. Figure 4E reveals a trend of decreasing RMSD values as CC values increase, suggesting that our CC energy term effectively directs the ligand toward the correct conformation. Furthermore, we also investigated the impact of density map resolution on the performance of DockEM and the CC, as shown in Supplementary File S5. We observed that the performance of DockEM decreases as the resolution of the density map decreases, which is expected. As the resolution of the density map lowers, the amount of pose information that can be extracted from the map also diminishes. This leads to a reduction in the number of sampling points within the sampling space, ultimately lowering the probability of obtaining reasonable conformations when using low-resolution density maps.

We also test our method on density map with resolution range from 10 Å to 15 Å the results can be found in the Supplementary File S7. It can be observed that the performance of our method declines when using density maps with resolutions above 10 Å, but it still comparable or better than other methods (as shown in Supplementary File S8). The average RMSD for these 10 cases was 2.39 Å for DockEM, 2.38 Å for CB-Dock2, 2.72 Å for ChemEM, 2.62 Å for EMERALD, and 2.91 Å for EDock. If the resolution is very low (>15 Å), this leads to the ligand’s density map being nearly indistinguishable from noise. Even if the density map corresponds to the ligand region, the low resolution means that the local density map would not contain much effective pose information.

**Figure 5:**
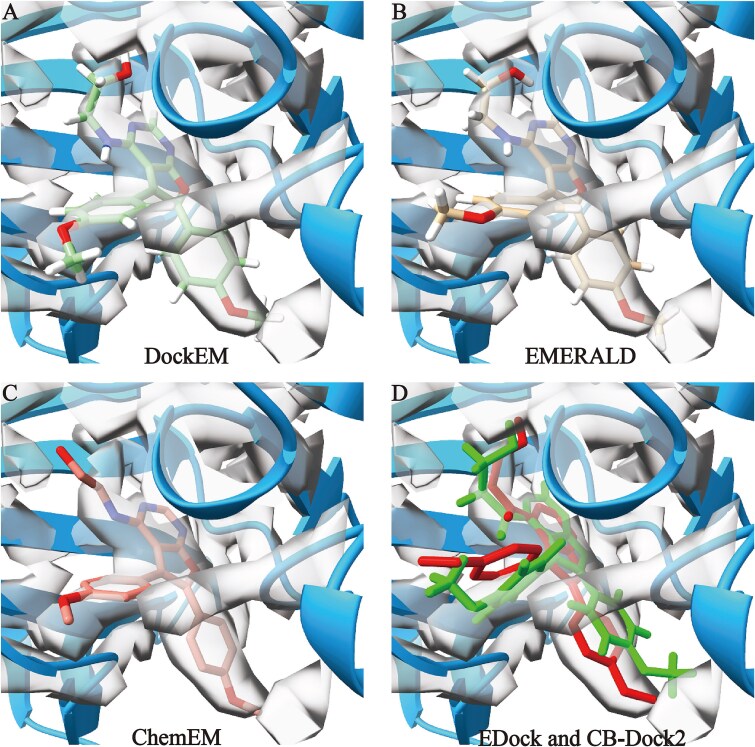
Comparison of ligand fitting to the local density map using five different docking methods for the protein–ligand complex (2br1A_BS01_PFP). The resolution of thr local decsity map is 3.69 Å, while the AF2-predicted protein model with a TM-score of 0.95. (A), (B), and (C) represent the ligand structures obtained by DockEM (RMSD of 0.43 Å and a CC value of 0.438), EMERALD (RMSD of 0.57 Å and a CC value of 0.422), and ChemEM (RMSD of 0.82 Å and a CC value of 0.411). (D) Represent the ligand structures obtained by CB-Dock2 (RMSD of 1.22 Å and a CC value of 0.344) and EDock (RMSD of 1.33 Å and a CC value of 0.325).

To further assess the fitting accuracy of ligand structures obtained from various docking methods relative to the local density map, we present a representative protein–ligand complex (PDB ID: 2br1A_BS01_PFP) in Fig. 5. Here, the gray region represents the local density map generated by DockEM at an initial resolution of 3.69 Å, while the blue region shows the predicted receptor protein model, which has a TM-score of 0.95. Figure 5A illustrates that the ligand structure generated by our DockEM method aligns closely with the local density map, achieving an RMSD of 0.49 Å and a cross-correlation (CC) value of 0.438. Figure 5B and C shows ligand structures obtained from the EMERALD and ChemEM, which fits reasonably well within the local density map, yielding a CC value of 0.422 and an RMSD of 0.57 Å for EMERALD and a CC value of 0.411 and an RMSD of 0.82 Å for ChemEM. In this case, only a small portion of the ligand extends beyond the local density map. In contrast, Fig. 5D depicts the ligand structures obtained from the EDock and CB-Dock2 methods, respectively, which display poor alignment with the local density map. Portions of these ligands fall outside the mapped density, resulting in CC and RMSD values of 0.325 and 1.33 Å for CB-Dock2, and 0.344 and 1.22 Å for EDock. Density maps are not used in either of these two methods. Here, they have included the density maps to facilitate a clearer comparison.

### Application to experimental density maps

**Figure 6:**
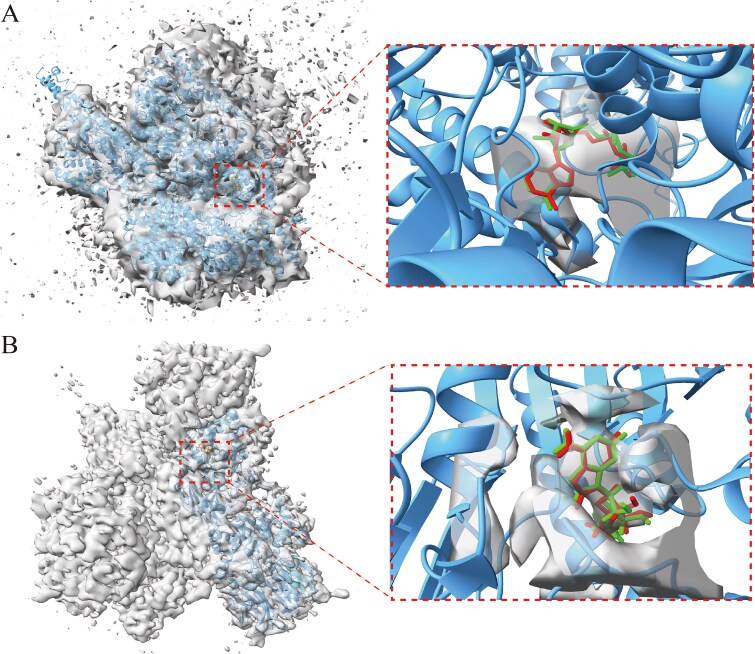
Docking results of ligands using DockEM in two experimental density maps. (A) Low-resolution experimental density map (ID: EMD-14083) from the EMDB, with a resolution of 7.0 Å. The RMSD of the docked ligand is 0.90 Å, and the CC value with the local density map is 0.43. (B) Medium-resolution experimental density map (ID: EMD-27078) from the EMDB, with a resolution of 3.14 Å. The RMSD of the docked ligand is 0.40 Å, and the CC value with the local density map is 0.47.

We validated our method using two experimental density maps, with the docking results presented in Fig. 6. The first experimental density map, characterized by low resolution, is from the 26S proteasome WT-Ubp6-UbVS complex in the si state (EMDB ID: EMD-14083, resolution 7.0 Å; corresponding fitted structure PDB ID: 7qo4). After docking with our method, we achieved an RMSD of 0.90 Å and a CC value of 0.43. The green ligand structure in Fig. 6A illustrates the result from our docking approach. The second experimental density map, which is of medium resolution, originates from the complex of PRMT5 and MEP50 in the presence of MTA and a novel inhibitor (11-2F) (EMDB ID: EMD-27078; resolution 3.14 Å; corresponding fitted structure PDB ID: 8cyi). In this case, our method yielded an RMSD of 0.40 Å and a CC value of 0.47, with the green ligand structure depicted in Fig. 6B. It is worth noting that in the second experiment’s density map, the ligand we docked is a pharmacophore from virtual screen leads to a specific inhibitor (11-2F). 11-2F can synergistically inhibit PRMT5 (an oncogenic target for various cancer types) by cooperating with MTA, a co-factor analog accumulated in MTAP−/− cells [41]. After performing docking with our method, a successful docking was achieved, yielding a ligand structure with an RMSD of 0.40 Å. This result provides insights into the ligand’s binding pose, which can be used to redesign new analogs that effectively synergize with MAT, thereby improving the catalytic efficiency of PRMT5 inhibition. This case illustrates how DockEM utilizes medium- to low-resolution density maps to dock ligands and address important real-world biological problems. These results demonstrate that our method is not only effective for simulated density maps but also performs well when applied to real experimental density maps.

## Conclusion

In this study, we present DockEM, an updated method designed to refine protein–ligand docking poses by incorporating information from cryo-EM density maps into the docking process. We conducted a thorough evaluation of DockEM on a test set of 121 protein–ligand complexes. The average RMSD achieved by DockEM was 1.87 Å, with successful docking of 110 ligands, resulting in a success rate of 90.9%. We also compared DockEM to several other docking programs, including EDock, ChemEM, EMERALD, and CB-Dock2. The results show that DockEM outperforms these programs in molecular docking, likely due to the advantages gained from integrating medium to low-resolution cryo-EM density map data. Furthermore, DockEM was tested on real experimental density maps, and its docking accuracy suggests promising potential for addressing key biological challenges in practical applications. However, the use of low-resolution cryo-EM density maps (with resolutions greater than 10 Å) presents a significant challenge.

Moreover, accurately localizing the ligand’s docking position, selecting appropriate parameters for the energy function, and ensuring the precision of cryo-EM density maps add complexity to the global optimization of protein–ligand docking within DockEM. To improve DockEM’s performance in future studies, we suggest several avenues for further investigation: (i) Developing methods to more precisely localize the ligand within the entire density map; (ii) Integrating deep learning frameworks for parameter selection to help identify optimal values; and (iii) Exploring optimization algorithms beyond REMC to accelerate the docking process. Our current approach, which uses cryo-EM density maps to elucidate atomic-level interactions, has demonstrated potential for structure-based drug design. Additionally, this approach could provide valuable insights to support future advancements in protein–peptide docking.

**Key Points**

- Advanced method for protein–ligand docking using cryo-EM density maps.
- Identify the local density map containing the ligand region from the overall density map to determine the precise docking position of the ligand on the protein.
- Physics- and knowledge-based energy functions, integrated with Cryo-EM density map data, were used to optimize the ligand structure in the REMC simulation.
- Significant ability to refine ligand on the local density maps.

## Supplementary Material

SI_bbaf091

## Data Availability

All data needed to evaluate the conclusions are present in the paper and the Supplementary Materials. The additional data and code related to this paper can be downloaded from https://github.com/Hsfssfwa/DockEM. DockEM is primarily implemented in C++ and does not require the use of a GPU when applying our method. The typical runtime for a molecular docking task using DockEM is approximately 60 min, although larger ligand molecules will increase the runtime. Detailed usage instructions for DockEM can be found in the README section of the code files.
