# Supplementary material for: DockEM: an enhanced method for atomic-scale protein–ligand docking refinement leveraging low-to-medium resolution cryo-EM density maps
Source: Brief Bioinform. 2025 Mar 10;26(2):bbaf091. doi: 10.1093/bib/bbaf091 (PMC11891657; doi:10.1093/bib/bbaf091)
Supplement: SI_bbaf091 [file si_bbaf091.docx]

**DockEM: An Enhanced Protocol for Atomic-Scale Protein-Ligand Docking Refinement Leveraging Low-to-Medium Resolution Cryo-EM Density Maps**

Jing Zou^1, +^, Wenyi Zhang^3, +^, Jun Hu^2,^ *, Xiaogen Zhou^1,^ *, Biao Zhang^2,^ *^, +^

^1^College of Information Engineering, Zhejiang University of Technology,

Hangzhou, Zhejiang 310023, China.

^2^Chinese Academy of Medical Sciences Suzhou Institute of Systems Medicine, Suzhou, Jiangsu, 215123, China.

^3^Westlake AI Therapeutics Lab, Westlake Laboratory of Life Sciences and Biomedicine, 18 Shilongshan Road, Hangzhou, Zhejiang 310024, China.

^+^Jing Zou, Wenyi Zhang and Biao Zhang contributed equally to this work.

* Corresponding authors: Biao Zhang, Suzhou Institute of Systems Medicine, Chinese Academy of Medical Sciences & Peking Union Medical College, Suzhou 215123, China. E-mail: zhangb@ism.cams.cn; Xiaogen Zhou, College of information Engineering, Zhejiang University of Technology, 288 Liuhe Road, Liuxia Street, Xihu District, Hangzhou 310023, China. Tel:15257118077; E-mail: zxg@zjut.edu.cn; Jun Hu, Suzhou Institute of Systems Medicine, Chinese Academy of Medical Sciences & Peking Union Medical College, Suzhou 215123, China. E-mail: hj@ism.cams.cn

**Supplementary File 1** Resolution of All Simulated Density Maps

| **ID** | **Resolution (Å)** | **ID** | **Resolution (Å)** |
| --- | --- | --- | --- |
| 1afkA_BS01_PAP | 8.21 | 1xm6A_BS02_5RM | 6.74 |
| 1arcA_BS01_TCK | 6.27 | 1xpyC_BS02_NLQ | 7.45 |
| 1azmA_BS02_AZM | 4.29 | 1xtbA_BS01_S6P | 9.00 |
| 1bs1A_BS02_ADP | 7.28 | 1ygcH_BS03_905 | 8.34 |
| 1c3jA_BS01_UDP | 8.47 | 1zu0A_BS01_CBS | 5.34 |
| 1cetA_BS01_CLQ | 5.39 | 2b99A_BS01_RDL | 5.32 |
| 1d4pB_BS03_BPP | 3.29 | 2b99A_BS03_RDL | 9.46 |
| 1e4gT_BS01_ATP | 6.14 | 2bawA_BS03_VCA | 5.96 |
| 1e5qA_BS01_NDP | 9.42 | 2br1A_BS01_PFP | 3.69 |
| 1f4eA_BS02_TPR | 5.82 | 2bsmA_BS01_BSM | 4.80 |
| 1f4fA_BS01_TP3 | 6.93 | 2cwhB_BS02_NDP | 5.09 |
| 1f4gA_BS01_TP4 | 3.25 | 2dkcA_BS01_16G | 5.04 |
| 1f7bA_BS01_NAU | 9.89 | 2dyaB_BS01_ADP | 8.64 |
| 1fkgA_BS01_SB3 | 5.54 | 2e9zA_BS04_UTP | 4.01 |
| 1foaA_BS02_UD1 | 5.06 | 2f9wA_BS01_PAU | 6.18 |
| 1g4oA_BS02_BSB | 4.83 | 2fk8A_BS01_SAM | 5.73 |
| 1g6cA_BS04_TZP | 9.06 | 2g25A_BS01_TDK | 5.14 |
| 1goqA_BS02_XYP | 7.44 | 2g25A_BS03_TDK | 4.54 |
| 1gpkA_BS01_HUP | 5.74 | 2gfxA_BS01_PMN | 4.56 |
| 1i7lA_BS01_ATP | 8.97 | 2ggaA_BS02_GPJ | 9.03 |
| 1if7A_BS02_SBR | 8.59 | 2gwhA_BS01_A3P | 6.61 |
| 1ixnA_BS01_DXP | 9.22 | 2hblA_BS03_AMP | 5.55 |
| 1k3uA_BS01_IAD | 3.26 | 2hixA_BS01_ATP | 3.73 |
| 1kgzB_BS03_PRP | 9.10 | 2ioaA_BS04_ADP | 5.46 |
| 1lspA_BS01_BUL | 5.62 | 2irxA_BS02_GTP | 6.28 |
| 1lzsA_BS02_NAG | 7.30 | 2jbtA_BS02_4HP | 8.95 |
| 1mbzA_BS03_IOT | 3.95 | 2ovdA_BS01_DAO | 4.36 |
| 1mkaA_BS02_DAC | 8.92 | 2q6vA_BS01_UDP | 3.68 |
| 1mmbA_BS05_BAT | 4.57 | 2qzzA_BS02_EMF | 7.20 |
| 1mq6A_BS02_XLD | 8.00 | 2rjcA_BS01_MES | 4.46 |
| 1mu0A_BS01_PHK | 7.11 | 2rjcA_BS02_MES | 9.78 |
| 1mxiA_BS01_SAH | 7.85 | 2rkmA_BS01_III | 4.49 |
| 1n46A_BS01_PFA | 7.31 | 2simA_BS01_DAN | 6.67 |
| 1navA_BS01_IH5 | 4.67 | 2uyqA_BS01_SAM | 9.97 |
| 1nhvA_BS01_154 | 7.35 | 2vkmA_BS01_BSD | 9.06 |
| 1o26B_BS01_UMP | 3.82 | 2wn7A_BS01_NAD | 9.16 |
| 1o26B_BS04_UMP | 8.47 | 3a0tA_BS01_ADP | 8.12 |
| 1onhA_BS01_WY4 | 9.65 | 3adpA_BS01_NAI | 3.83 |
| 1oxmA_BS01_TC4 | 9.58 | 3b3fA_BS01_SAH | 8.54 |
| 1oytH_BS04_FSN | 5.59 | 3b4yA_BS02_FLC | 8.68 |
| 1p77A_BS01_ATR | 8.56 | 3bynA_BS01_RAF | 7.8 |
| 1pfkA_BS03_ADP | 7.23 | 3cagA_BS01_ARG | 8.07 |
| 1pkkB_BS01_DCP | 8 | 3cpaA_BS01_III | 7.58 |
| 1ppcE_BS01_MID | 9.11 | 3du4A_BS02_KAP | 6.25 |
| 1pphE_BS02_0ZG | 4.42 | 3e3sA_BS04_I3C | 7.52 |
| 1q41A_BS01_IXM | 7.57 | 3efvA_BS01_NAD | 6.8 |
| 1ql9A_BS02_ZEN | 9.75 | 3fwrA_BS01_ADP | 3.37 |
| 1rzuA_BS01_ADP | 8.83 | 3gidA_BS01_S1A | 5.95 |
| 1sbyA_BS01_NAD | 10 | 3gpoA_BS01_APR | 7.72 |
| 1sqfA_BS01_SAM | 3.97 | 3gqkA_BS01_ATP | 6.24 |
| 1sz3A_BS01_GNP | 8.18 | 3h39A_BS01_ATP | 4.47 |
| 1theA_BS01_0E6 | 4.00 | 3hvoA_BS01_VGI | 6.75 |
| 1thlA_BS06_0DB | 9.56 | 3idoA_BS01_EPE | 3.61 |
| 1towA_BS01_CRZ | 7.71 | 3iiqA_BS02_JZA | 6.54 |
| 1uvtH_BS02_I48 | 7.02 | 3jynA_BS01_NDP | 8.87 |
| 1v0yA_BS01_HI5 | 6.02 | 3ldkA_BS01_SUC | 6.23 |
| 1v3sA_BS02_ATP | 5.46 | 3ll3A_BS01_ATP | 7.05 |
| 1wopA_BS01_FFO | 8.61 | 3ll3A_BS03_XUL | 3.2 |
| 1wopA_BS02_FFO | 3.17 | 3stdA_BS01_MQ0 | 5.11 |
| 1wxiA_BS03_AMP | 8.04 | 7dfrA_BS02_NAP | 4.18 |
| 1x7pA_BS01_SAM | 6.23 |  |  |

**Supplementary File 2** Lists the TM-scores of each protein and the RMSD of each initial ligand

| **Case ID** | **TM-score** | **RMSD(Å)** | **Case ID** | **TM-score** | **RMSD(Å)** |
| --- | --- | --- | --- | --- | --- |
| 1afkA_BS01_PAP | 0.995 | 51.70 | 1xm6A_BS02_5RM | 0.959 | 36.77 |
| 1arcA_BS01_TCK | 0.997 | 21.63 | 1xpyC_BS02_NLQ | 0.992 | 71.41 |
| 1azmA_BS02_AZM | 0.990 | 43.61 | 1xtbA_BS01_S6P | 0.996 | 24.91 |
| 1bs1A_BS02_ADP | 0.993 | 25.40 | 1ygcH_BS03_905 | 0.985 | 58.34 |
| 1c3jA_BS01_UDP | 0.978 | 29.99 | 1zu0A_BS01_CBS | 0.975 | 43.79 |
| 1cetA_BS01_CLQ | 0.985 | 47.21 | 2b99A_BS01_RDL | 0.977 | 24.05 |
| 1d4pB_BS03_BPP | 0.995 | 34.72 | 2b99A_BS03_RDL | 0.977 | 37.84 |
| 1e4gT_BS01_ATP | 0.983 | 54.32 | 2bawA_BS03_VCA | 0.974 | 65.36 |
| 1e5qA_BS01_NDP | 0.984 | 80.07 | 2br1A_BS01_PFP | 0.951 | 30.30 |
| 1f4eA_BS02_TPR | 0.986 | 116.90 | 2bsmA_BS01_BSM | 0.978 | 45.26 |
| 1f4fA_BS01_TP3 | 0.992 | 41.68 | 2cwhB_BS02_NDP | 0.991 | 87.97 |
| 1f4gA_BS01_TP4 | 0.991 | 48.63 | 2dkcA_BS01_16G | 0.995 | 32.61 |
| 1f7bA_BS01_NAU | 0.996 | 29.42 | 2dyaB_BS01_ADP | 0.939 | 70.26 |
| 1fkgA_BS01_SB3 | 0.991 | 41.44 | 2e9zA_BS04_UTP | 0.991 | 39.81 |
| 1foaA_BS02_UD1 | 0.991 | 21.87 | 2f9wA_BS01_PAU | 0.984 | 68.47 |
| 1g4oA_BS02_BSB | 0.995 | 24.81 | 2fk8A_BS01_SAM | 0.954 | 34.89 |
| 1g6cA_BS04_TZP | 0.998 | 45.48 | 2g25A_BS01_TDK | 0.998 | 92.57 |
| 1goqA_BS02_XYP | 0.998 | 16.96 | 2g25A_BS03_TDK | 0.998 | 75.00 |
| 1gpkA_BS01_HUP | 0.998 | 97.53 | 2gfxA_BS01_PMN | 0.995 | 72.07 |
| 1i7lA_BS01_ATP | 0.985 | 63.33 | 2ggaA_BS02_GPJ | 0.994 | 184.83 |
| 1if7A_BS02_SBR | 0.996 | 24.87 | 2gwhA_BS01_A3P | 0.983 | 37.30 |
| 1ixnA_BS01_DXP | 0.966 | 21.08 | 2hblA_BS03_AMP | 0.979 | 84.75 |
| 1k3uA_BS01_IAD | 0.987 | 63.23 | 2hixA_BS01_ATP | 0.745 | 44.71 |
| 1kgzB_BS03_PRP | 0.997 | 47.45 | 2ioaA_BS04_ADP | 0.995 | 24.67 |
| 1lspA_BS01_BUL | 0.994 | 27.03 | 2irxA_BS02_GTP | 0.992 | 36.72 |
| 1lzsA_BS02_NAG | 0.992 | 27.27 | 2jbtA_BS02_4HP | 0.980 | 108.13 |
| 1mbzA_BS03_IOT | 0.976 | 121.83 | 2ovdA_BS01_DAO | 0.965 | 51.80 |
| 1mkaA_BS02_DAC | 0.994 | 22.46 | 2q6vA_BS01_UDP | 0.980 | 83.54 |
| 1mmbA_BS05_BAT | 0.991 | 86.09 | 2qzzA_BS02_EMF | 0.990 | 33.17 |
| 1mq6A_BS02_XLD | 0.993 | 22.16 | 2rjcA_BS01_MES | 0.985 | 58.82 |
| 1mu0A_BS01_PHK | 0.991 | 15.30 | 2rjcA_BS02_MES | 0.985 | 32.60 |
| 1mxiA_BS01_SAH | 0.961 | 28.39 | 2rkmA_BS01_III | 0.996 | 20.81 |
| 1n46A_BS01_PFA | 0.972 | 16.30 | 2simA_BS01_DAN | 0.995 | 81.68 |
| 1navA_BS01_IH5 | 0.941 | 55.54 | 2uyqA_BS01_SAM | 0.965 | 24.22 |
| 1nhvA_BS01_154 | 0.992 | 112.65 | 2vkmA_BS01_BSD | 0.969 | 32.22 |
| 1o26B_BS01_UMP | 0.982 | 107.96 | 2wn7A_BS01_NAD | 0.988 | 36.66 |
| 1o26B_BS04_UMP | 0.982 | 100.58 | 3a0tA_BS01_ADP | 0.987 | 42.73 |
| 1onhA_BS01_WY4 | 0.980 | 32.98 | 3adpA_BS01_NAI | 0.988 | 38.47 |
| 1oxmA_BS01_TC4 | 0.994 | 51.21 | 3b3fA_BS01_SAH | 0.992 | 144.07 |
| 1oytH_BS04_FSN | 0.994 | 27.75 | 3b4yA_BS02_FLC | 0.992 | 55.79 |
| 1p77A_BS01_ATR | 0.985 | 45.32 | 3bynA_BS01_RAF | 0.999 | 58.13 |
| 1pfkA_BS03_ADP | 0.990 | 23.50 | 3cagA_BS01_ARG | 0.993 | 34.46 |
| 1pkkB_BS01_DCP | 0.981 | 73.14 | 3cpaA_BS01_III | 0.998 | 36.53 |
| 1ppcE_BS01_MID | 0.995 | 22.53 | 3du4A_BS02_KAP | 0.995 | 23.78 |
| 1pphE_BS02_0ZG | 0.995 | 25.56 | 3e3sA_BS04_I3C | 0.998 | 51.12 |
| 1q41A_BS01_IXM | 0.981 | 52.29 | 3efvA_BS01_NAD | 0.994 | 112.34 |
| 1ql9A_BS02_ZEN | 0.997 | 83.53 | 3fwrA_BS01_ADP | 0.963 | 51.93 |
| 1rzuA_BS01_ADP | 0.935 | 85.38 | 3gidA_BS01_S1A | 0.963 | 47.89 |
| 1sbyA_BS01_NAD | 0.994 | 40.30 | 3gpoA_BS01_APR | 0.978 | 40.89 |
| 1sqfA_BS01_SAM | 0.993 | 16.63 | 3gqkA_BS01_ATP | 0.949 | 28.58 |
| 1sz3A_BS01_GNP | 0.986 | 41.37 | 3h39A_BS01_ATP | 0.953 | 87.28 |
| 1theA_BS01_0E6 | 0.996 | 89.16 | 3hvoA_BS01_VGI | 0.993 | 97.52 |
| 1thlA_BS06_0DB | 0.998 | 53.90 | 3idoA_BS01_EPE | 0.983 | 95.11 |
| 1towA_BS01_CRZ | 0.989 | 20.81 | 3iiqA_BS02_JZA | 0.982 | 16.22 |
| 1uvtH_BS02_I48 | 0.989 | 78.57 | 3jynA_BS01_NDP | 0.995 | 20.72 |
| 1v0yA_BS01_HI5 | 0.998 | 17.18 | 3ldkA_BS01_SUC | 0.999 | 106.99 |
| 1v3sA_BS02_ATP | 0.956 | 23.90 | 3ll3A_BS01_ATP | 0.992 | 37.66 |
| 1wopA_BS01_FFO | 0.994 | 28.96 | 3ll3A_BS03_XUL | 0.992 | 25.90 |
| 1wopA_BS02_FFO | 0.994 | 37.37 | 3stdA_BS01_MQ0 | 0.990 | 44.54 |
| 1wxiA_BS03_AMP | 0.989 | 77.42 | 7dfrA_BS02_NAP | 0.986 | 35.72 |
| 1x7pA_BS01_SAM | 0.968 | 76.30 |  |  |  |

**Supplementary File 3** The docking result: 1) The result of distance between the refinement ligand center and native ligand center (refinement); 2) The result of distance between the ligand center of rigid-body docking and native ligand center (rigid-body docking); 3) The result of distance between the Binding site center and native ligand center (Binding sites center distance).

| **Case ID** | **Refinement (Å)** | **rigid-body docking (Å)** | **Binding sites center distance (Å)** |
| --- | --- | --- | --- |
| 1afkA_BS01_PAP | 0.56 | 0.61 | 5.02 |
| 1arcA_BS01_TCK | 0.28 | 0.66 | 3.42 |
| 1azmA_BS02_AZM | 0.28 | 0.71 | 4.64 |
| 1bs1A_BS02_ADP | 1.05 | 3.52 | 7.27 |
| 1c3jA_BS01_UDP | 0.17 | 0.40 | 2.33 |
| 1cetA_BS01_CLQ | 2.15 | 2.14 | 5.89 |
| 1d4pB_BS03_BPP | 4.02 | 3.62 | 6.01 |
| 1e4gT_BS01_ATP | 0.72 | 1.69 | 8.67 |
| 1e5qA_BS01_NDP | 1.58 | 2.16 | 1.90 |
| 1f4eA_BS02_TPR | 0.70 | 0.71 | 7.65 |
| 1f4fA_BS01_TP3 | 0.56 | 0.54 | 4.10 |
| 1f4gA_BS01_TP4 | 0.51 | 3.99 | 4.79 |
| 1f7bA_BS01_NAU | 1.73 | 2.15 | 4.04 |
| 1fkgA_BS01_SB3 | 1.42 | 1.79 | 5.16 |
| 1foaA_BS02_UD1 | 0.78 | 0.76 | 2.55 |
| 1g4oA_BS02_BSB | 0.38 | 0.67 | 3.36 |
| 1g6cA_BS04_TZP | 0.36 | 0.72 | 1.79 |
| 1goqA_BS02_XYP | 0.59 | 1.06 | 3.27 |
| 1gpkA_BS01_HUP | 0.69 | 0.62 | 3.47 |
| 1i7lA_BS01_ATP | 0.80 | 2.13 | 1.81 |
| 1if7A_BS02_SBR | 0.79 | 2.13 | 8.42 |
| 1ixnA_BS01_DXP | 1.23 | 2.47 | 4.58 |
| 1k3uA_BS01_IAD | 0.57 | 0.65 | 7.97 |
| 1kgzB_BS03_PRP | 1.59 | 0.99 | 3.01 |
| 1lspA_BS01_BUL | 0.97 | 4.43 | 5.83 |
| 1lzsA_BS02_NAG | 0.80 | 0.89 | 4.47 |
| 1mbzA_BS03_IOT | 1.74 | 2.57 | 2.50 |
| 1mkaA_BS02_DAC | 0.96 | 1.19 | 4.08 |
| 1mmbA_BS05_BAT | 0.60 | 0.80 | 3.70 |
| 1mq6A_BS02_XLD | 1.34 | 1.51 | 4.25 |
| 1mu0A_BS01_PHK | 0.94 | 2.46 | 6.28 |
| 1mxiA_BS01_SAH | 0.49 | 0.98 | 1.26 |
| 1n46A_BS01_PFA | 0.83 | 0.81 | 2.61 |
| 1navA_BS01_IH5 | 1.07 | 0.71 | 6.28 |
| 1nhvA_BS01_154 | 0.34 | 0.51 | 6.98 |
| 1o26B_BS01_UMP | 0.58 | 0.28 | 4.37 |
| 1o26B_BS04_UMP | 0.60 | 1.24 | 5.80 |
| 1onhA_BS01_WY4 | 0.91 | 0.38 | 2.04 |
| 1oxmA_BS01_TC4 | 0.71 | 2.22 | 5.82 |
| 1oytH_BS04_FSN | 1.26 | 1.73 | 2.81 |
| 1p77A_BS01_ATR | 0.89 | 2.76 | 7.56 |
| 1pfkA_BS03_ADP | 0.91 | 1.07 | 2.39 |
| 1pkkB_BS01_DCP | 0.91 | 3.33 | 10.82 |
| 1ppcE_BS01_MID | 2.52 | 2.52 | 5.42 |
| 1pphE_BS02_0ZG | 1.07 | 2.54 | 8.41 |
| 1q41A_BS01_IXM | 0.52 | 0.70 | 2.91 |
| 1ql9A_BS02_ZEN | 1.49 | 3.47 | 5.54 |
| 1rzuA_BS01_ADP | 1.59 | 2.46 | 6.22 |
| 1sbyA_BS01_NAD | 1.40 | 2.09 | 2.55 |
| 1sqfA_BS01_SAM | 0.31 | 3.43 | 5.59 |
| 1sz3A_BS01_GNP | 1.21 | 2.41 | 7.33 |
| 1theA_BS01_0E6 | 1.48 | 1.67 | 6.22 |
| 1thlA_BS06_0DB | 0.69 | 1.19 | 3.60 |
| 1towA_BS01_CRZ | 1.30 | 0.85 | 5.88 |
| 1uvtH_BS02_I48 | 0.61 | 1.03 | 4.46 |
| 1v0yA_BS01_HI5 | 0.78 | 1.30 | 2.11 |
| 1v3sA_BS02_ATP | 0.57 | 0.50 | 3.52 |
| 1wopA_BS01_FFO | 1.11 | 1.47 | 1.79 |
| 1wopA_BS02_FFO | 0.40 | 0.37 | 5.76 |
| 1wxiA_BS03_AMP | 0.26 | 0.37 | 3.70 |
| 1x7pA_BS01_SAM | 0.82 | 1.05 | 3.78 |
| 1xm6A_BS02_5RM | 0.33 | 1.08 | 4.57 |
| 1xpyC_BS02_NLQ | 0.51 | 2.18 | 6.79 |
| 1xtbA_BS01_S6P | 0.21 | 0.13 | 6.07 |
| 1ygcH_BS03_905 | 1.27 | 1.93 | 5.68 |
| 1zu0A_BS01_CBS | 1.02 | 1.13 | 2.17 |
| 2b99A_BS01_RDL | 0.59 | 1.70 | 6.59 |
| 2b99A_BS03_RDL | 0.72 | 1.35 | 1.44 |
| 2bawA_BS03_VCA | 1.24 | 0.80 | 3.98 |
| 2br1A_BS01_PFP | 0.37 | 0.84 | 4.64 |
| 2bsmA_BS01_BSM | 0.73 | 0.61 | 4.51 |
| 2cwhB_BS02_NDP | 1.06 | 2.49 | 5.25 |
| 2dkcA_BS01_16G | 0.40 | 1.32 | 3.96 |
| 2dyaB_BS01_ADP | 1.38 | 1.32 | 3.48 |
| 2e9zA_BS04_UTP | 1.13 | 1.74 | 12.55 |
| 2f9wA_BS01_PAU | 0.83 | 0.71 | 3.04 |
| 2fk8A_BS01_SAM | 1.22 | 3.10 | 3.23 |
| 2g25A_BS01_TDK | 0.38 | 0.85 | 7.37 |
| 2g25A_BS03_TDK | 1.81 | 3.67 | 7.39 |
| 2gfxA_BS01_PMN | 1.40 | 2.46 | 5.23 |
| 2ggaA_BS02_GPJ | 1.75 | 1.74 | 4.59 |
| 2gwhA_BS01_A3P | 0.61 | 1.40 | 2.49 |
| 2hblA_BS03_AMP | 0.48 | 2.22 | 9.74 |
| 2hixA_BS01_ATP | 0.50 | 2.82 | 4.16 |
| 2ioaA_BS04_ADP | 0.48 | 0.87 | 7.50 |
| 2irxA_BS02_GTP | 0.38 | 1.57 | 2.22 |
| 2jbtA_BS02_4HP | 0.20 | 2.13 | 6.55 |
| 2ovdA_BS01_DAO | 0.94 | 0.92 | 3.94 |
| 2q6vA_BS01_UDP | 0.52 | 1.93 | 0.72 |
| 2qzzA_BS02_EMF | 0.95 | 1.51 | 3.34 |
| 2rjcA_BS01_MES | 0.34 | 0.16 | 4.38 |
| 2rjcA_BS02_MES | 0.95 | 1.01 | 2.70 |
| 2rkmA_BS01_III | 0.56 | 1.76 | 2.80 |
| 2simA_BS01_DAN | 0.49 | 3.57 | 8.51 |
| 2uyqA_BS01_SAM | 0.96 | 1.20 | 5.16 |
| 2vkmA_BS01_BSD | 1.42 | 5.99 | 7.48 |
| 2wn7A_BS01_NAD | 1.23 | 1.35 | 4.02 |
| 3a0tA_BS01_ADP | 0.28 | 3.13 | 9.34 |
| 3adpA_BS01_NAI | 2.74 | 4.17 | 5.88 |
| 3b3fA_BS01_SAH | 0.29 | 1.29 | 2.01 |
| 3b4yA_BS02_FLC | 1.64 | 1.12 | 4.52 |
| 3bynA_BS01_RAF | 0.55 | 0.63 | 6.77 |
| 3cagA_BS01_ARG | 0.56 | 0.79 | 3.80 |
| 3cpaA_BS01_III | 0.47 | 0.78 | 1.92 |
| 3du4A_BS02_KAP | 0.36 | 0.96 | 9.72 |
| 3e3sA_BS04_I3C | 1.19 | 0.96 | 2.43 |
| 3efvA_BS01_NAD | 0.97 | 2.16 | 2.65 |
| 3fwrA_BS01_ADP | 0.73 | 2.11 | 12.97 |
| 3gidA_BS01_S1A | 2.15 | 2.39 | 5.25 |
| 3gpoA_BS01_APR | 4.25 | 4.07 | 2.22 |
| 3gqkA_BS01_ATP | 0.60 | 3.73 | 6.59 |
| 3h39A_BS01_ATP | 0.15 | 2.83 | 10.83 |
| 3hvoA_BS01_VGI | 0.85 | 0.85 | 6.67 |
| 3idoA_BS01_EPE | 0.32 | 0.97 | 8.39 |
| 3iiqA_BS02_JZA | 0.53 | 0.53 | 7.62 |
| 3jynA_BS01_NDP | 1.04 | 2.45 | 2.25 |
| 3ldkA_BS01_SUC | 0.29 | 0.90 | 6.37 |
| 3ll3A_BS01_ATP | 0.92 | 2.96 | 6.60 |
| 3ll3A_BS03_XUL | 0.25 | 0.52 | 5.79 |
| 3stdA_BS01_MQ0 | 0.86 | 3.54 | 11.92 |
| 7dfrA_BS02_NAP | 1.19 | 4.81 | 6.17 |

**Supplementary File 4** Performance Comparison of Different CC Calculation Methods

In DockEM, we implemented two methods for calculating the CC (cross-correlation) value: one directly calculates the CC between the ligand and the local density map, while the other calculates the CC between the ligand plus certain surrounding protein atoms and the local density map. These methods were tested on a dataset, with results shown in Fig S1A. The figure compares the performance of the two approaches: DockEM calculates the CC between only the ligand and the density map, whereas DockEM* includes additional surrounding protein atoms in the CC calculation. The average RMSD values achieved by these methods across the dataset were 1.87 Å for DockEM and 1.99 Å for DockEM*, with success rates of 90.9% and 86.7%, respectively. As shown in Fig S1B, the differences in performance between the two approaches are minimal. However, DockEM is significantly faster than DockEM*, which is expected given that calculating the CC for all ligand atoms alone requires less computational time than adding protein atoms as well. The total number of ligand atoms and the number of ligand-plus-protein atoms for all ligands in the dataset are detailed in Supplementary File 6. Consequently, we opted for the method that calculates the CC value between only the ligand and the density map.


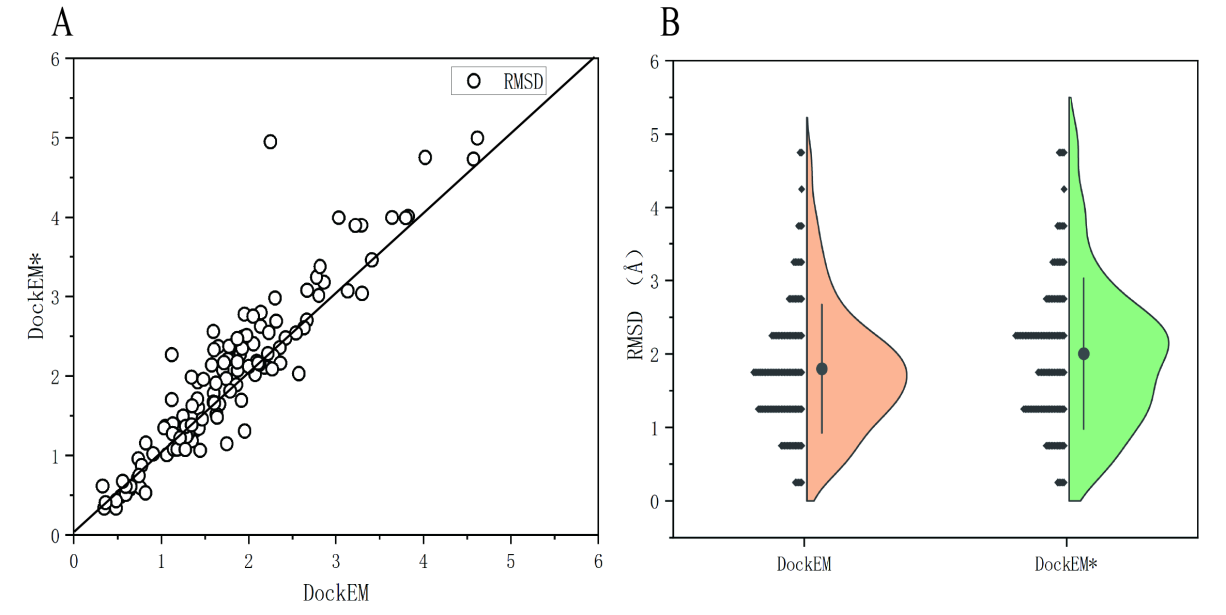


**Figure S1.** Shows the RMSD between the ligand structures obtained using two different CC calculation methods in DockEM and the native structure. DockEM computes the CC value between the ligand and the local density map, while DockEM* includes the ligand and surrounding protein atoms in the CC calculation. **A)** Scatter plot comparing the ligand RMSD results for DockEM and DockEM*. **B)** Box plot comparing the ligand RMSD for the two methods, with the orange box plot representing DockEM and the green box plot representing DockEM*.

**Supplementary File 5** The impact of density map resolution on performance

In DockEM, we compared the impact of density map resolution on the performance of our method. We divided the density maps used in the 121 cases into three resolution ranges: 3-6Å, 6-8Å, and 8-10Å. As shown in Fig S2A, in the 3-6Å resolution range, the average RMSD for all cases was 1.61Å, which is lower than the RMSD for the 6-8Å resolution range (1.68Å) and the 8-10Å resolution range (2.31Å). Additionally, we compared the ligand's correlation coefficient (CC) with the local density map across different resolution ranges, as shown in Fig S2B. In the 3-6Å range, the average CC for all cases was 0.78, higher than the CC of 0.75 in the 6-8Å range and 0.69 in the 8-10Å range. This shows that as the resolution of the density map decreases, the quality of the docked ligands also decreases. This is because, at lower resolutions, it becomes more difficult to accurately retrieve the ligand poses from the local density map.


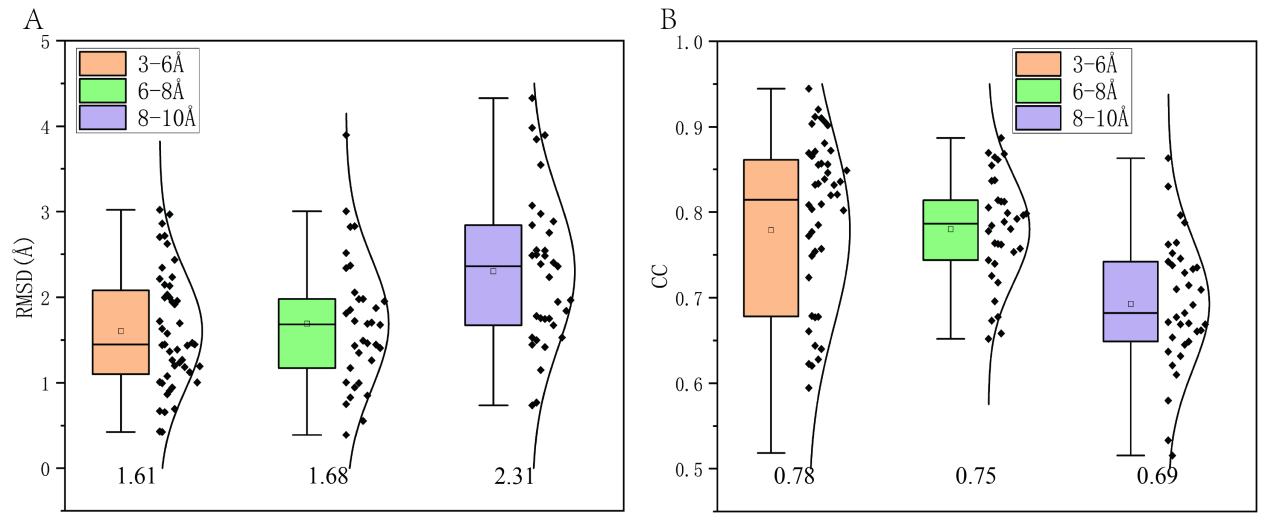


**Figure S2.** Comparison of performance across different resolution ranges of density maps. The orange section represents density maps in the 3-6 Å range, the green section represents those in the 6-8 Å range, and the purple section represents those in the 8-10 Å range. **A)** Box plot comparison of RMSD across three resolution ranges. The average RMSD for density maps in the 3-6 Å range is 1.61 Å, in the 6-8 Å range it is 1.68 Å, and in the 8-10 Å range it is 2.31 Å. **B)** Box plot comparison of CC values across three resolution ranges. The average CC for density maps in the 3-6 Å range is 0.78, in the 6-8 Å range it is 0.75, and in the 8-10 Å range it is 0.69.

**Supplementary File 6** The total number of atoms

| **ID** | **Num1^*^** | **Num2^+^** | **ID** | **Num1^*^** | **Num2^+^** |
| --- | --- | --- | --- | --- | --- |
| 1afkA_BS01_PAP | 42 | 456 | 1xm6A_BS02_5RM | 38 | 154 |
| 1arcA_BS01_TCK | 43 | 479 | 1xpyC_BS02_NLQ | 29 | 70 |
| 1azmA_BS02_AZM | 19 | 127 | 1xtbA_BS01_S6P | 29 | 150 |
| 1bs1A_BS02_ADP | 40 | 145 | 1ygcH_BS03_905 | 64 | 785 |
| 1c3jA_BS01_UDP | 36 | 405 | 1zu0A_BS01_CBS | 57 | 339 |
| 1cetA_BS01_CLQ | 49 | 249 | 2b99A_BS01_RDL | 35 | 92 |
| 1d4pB_BS03_BPP | 49 | 1176 | 2b99A_BS03_RDL | 35 | 60 |
| 1e4gT_BS01_ATP | 43 | 876 | 2bawA_BS03_VCA | 50 | 1332 |
| 1e5qA_BS01_NDP | 73 | 2001 | 2br1A_BS01_PFP | 50 | 864 |
| 1f4eA_BS02_TPR | 31 | 87 | 2bsmA_BS01_BSM | 45 | 812 |
| 1f4fA_BS01_TP3 | 43 | 1167 | 2cwhB_BS02_NDP | 69 | 594 |
| 1f4gA_BS01_TP4 | 53 | 922 | 2dkcA_BS01_16G | 33 | 196 |
| 1f7bA_BS01_NAU | 40 | 163 | 2dyaB_BS01_ADP | 39 | 148 |
| 1fkgA_BS01_SB3 | 68 | 479 | 2e9zA_BS04_UTP | 40 | 286 |
| 1foaA_BS02_UD1 | 64 | 951 | 2f9wA_BS01_PAU | 30 | 129 |
| 1g4oA_BS02_BSB | 32 | 276 | 2fk8A_BS01_SAM | 48 | 710 |
| 1g6cA_BS04_TZP | 22 | 154 | 2g25A_BS01_TDK | 61 | 332 |
| 1goqA_BS02_XYP | 20 | 33 | 2g25A_BS03_TDK | 61 | 403 |
| 1gpkA_BS01_HUP | 37 | 130 | 2gfxA_BS01_PMN | 58 | 1062 |
| 1i7lA_BS01_ATP | 43 | 866 | 2ggaA_BS02_GPJ | 17 | 25 |
| 1if7A_BS02_SBR | 47 | 405 | 2gwhA_BS01_A3P | 38 | 368 |
| 1ixnA_BS01_DXP | 22 | 47 | 2hblA_BS03_AMP | 35 | 117 |
| 1k3uA_BS01_IAD | 31 | 238 | 2hixA_BS01_ATP | 43 | 361 |
| 1kgzB_BS03_PRP | 30 | 150 | 2ioaA_BS04_ADP | 38 | 459 |
| 1lspA_BS01_BUL | 65 | 306 | 2irxA_BS02_GTP | 48 | 569 |
| 1lzsA_BS02_NAG | 57 | 330 | 2jbtA_BS02_4HP | 17 | 52 |
| 1mbzA_BS03_IOT | 62 | 1352 | 2ovdA_BS01_DAO | 36 | 510 |
| 1mkaA_BS02_DAC | 43 | 706 | 2q6vA_BS01_UDP | 36 | 225 |
| 1mmbA_BS05_BAT | 63 | 419 | 2qzzA_BS02_EMF | 33 | 125 |
| 1mq6A_BS02_XLD | 54 | 1050 | 2rjcA_BS01_MES | 26 | 82 |
| 1mu0A_BS01_PHK | 28 | 169 | 2rjcA_BS02_MES | 26 | 101 |
| 1mxiA_BS01_SAH | 45 | 409 | 2rkmA_BS01_III | 46 | 542 |
| 1n46A_BS01_PFA | 48 | 1420 | 2simA_BS01_DAN | 35 | 109 |
| 1navA_BS01_IH5 | 37 | 494 | 2uyqA_BS01_SAM | 28 | 128 |
| 1nhvA_BS01_154 | 56 | 737 | 2vkmA_BS01_BSD | 90 | 934 |
| 1o26B_BS01_UMP | 31 | 68 | 2wn7A_BS01_NAD | 70 | 983 |
| 1o26B_BS04_UMP | 31 | 75 | 3a0tA_BS01_ADP | 39 | 248 |
| 1onhA_BS01_WY4 | 32 | 236 | 3adpA_BS01_NAI | 70 | 410 |
| 1oxmA_BS01_TC4 | 61 | 408 | 3b3fA_BS01_SAH | 45 | 509 |
| 1oytH_BS04_FSN | 52 | 396 | 3b4yA_BS02_FLC | 16 | 47 |
| 1p77A_BS01_ATR | 41 | 203 | 3bynA_BS01_RAF | 64 | 623 |
| 1pfkA_BS03_ADP | 39 | 198 | 3cagA_BS01_ARG | 25 | 77 |
| 1pkkB_BS01_DCP | 42 | 247 | 3cpaA_BS01_III | 30 | 112 |
| 1ppcE_BS01_MID | 66 | 791 | 3du4A_BS02_KAP | 29 | 131 |
| 1pphE_BS02_0ZG | 56 | 372 | 3e3sA_BS04_I3C | 18 | 35 |
| 1q41A_BS01_IXM | 32 | 268 | 3efvA_BS01_NAD | 70 | 1177 |
| 1ql9A_BS02_ZEN | 61 | 299 | 3fwrA_BS01_ADP | 39 | 346 |
| 1rzuA_BS01_ADP | 39 | 271 | 3gidA_BS01_S1A | 81 | 726 |
| 1sbyA_BS01_NAD | 70 | 2907 | 3gpoA_BS01_APR | 57 | 1845 |
| 1sqfA_BS01_SAM | 48 | 477 | 3gqkA_BS01_ATP | 43 | 438 |
| 1sz3A_BS01_GNP | 47 | 547 | 3h39A_BS01_ATP | 43 | 404 |
| 1theA_BS01_0E6 | 68 | 1014 | 3hvoA_BS01_VGI | 41 | 716 |
| 1thlA_BS06_0DB | 63 | 770 | 3idoA_BS01_EPE | 35 | 139 |
| 1towA_BS01_CRZ | 32 | 185 | 3iiqA_BS02_JZA | 26 | 70 |
| 1uvtH_BS02_I48 | 49 | 1281 | 3jynA_BS01_NDP | 73 | 790 |
| 1v0yA_BS01_HI5 | 40 | 127 | 3ldkA_BS01_SUC | 45 | 209 |
| 1v3sA_BS02_ATP | 43 | 188 | 3ll3A_BS01_ATP | 43 | 522 |
| 1wopA_BS01_FFO | 52 | 1678 | 3ll3A_BS03_XUL | 20 | 30 |
| 1wopA_BS02_FFO | 50 | 1036 | 3stdA_BS01_MQ0 | 48 | 699 |
| 1wxiA_BS03_AMP | 36 | 190 | 7dfrA_BS02_NAP | 75 | 1247 |
| 1x7pA_BS01_SAM | 48 | 634 |  |  |  |

* represents the total number of atoms in the ligand.

+ represents the total number of atoms in the ligand plus a portion of the protein.

**Supplementary File 7** Summary of the RMSD values for the density map with resolution > 10 Å in DockEM

| **Case** | **Resolution(Å)** | **RMSD(Å)** | **Resolution(Å)** | **RMSD(Å)** |
| --- | --- | --- | --- | --- |
| 1azmA_BS02_AZM | 4.29 | 1.42 | 10.51 | 2.05 |
| 1f4eA_BS02_TPR | 5.82 | 1.59 | 12.59 | 2.46 |
| 1goqA_BS02_XYP | 7.44 | 0.64 | 13.84 | 2.18 |
| 1kgzB_BS03_PRP | 9.10 | 3.14 | 13.26 | 4.56 |
| 1n46A_BS01_PFA | 7.31 | 0.35 | 4.42 | 1.08 |
| 1x7pA_BS01_SAM | 6.23 | 1.35 | 12.76 | 2.14 |
| 2dyaB_BS01_ADP | 8.64 | 1.76 | 15.41 | 3.05 |
| 2ovdA_BS01_DAO | 4.36 | 1.13 | 10.99 | 2.39 |
| 3cpaA_BS01_III | 7.58 | 1.34 | 11.29 | 2.11 |
| 3stdA_BS01_MQ0 | 5.11 | 0.82 | 12.08 | 1.95 |

**Supplementary File 8** Summary of the RMSD values for the different methods with density map resolution > 10 Å.

| **Method** | **DockEM**  **(Å)** | **CB-Dock2**  **(Å)** | **ChemEM**  **(Å)** | **EDock**  **(Å)** | **EMERALD**  **(Å)** |
| --- | --- | --- | --- | --- | --- |
| 1azmA_BS02_AZM | 2.05 | 2.18 | 2.44 | 1.76 | 2.83 |
| 1f4eA_BS02_TPR | 2.46 | 4.19 | 5.74 | 4.55 | 2.21 |
| 1goqA_BS02_XYP | 2.18 | 2.78 | 1.12 | 4.13 | 2.66 |
| 1kgzB_BS03_PRP | 4.56 | 4.67 | 4.45 | 6.44 | 4.89 |
| 1n46A_BS01_PFA | 1.08 | 0.44 | 1.99 | 0.56 | 0.95 |
| 1x7pA_BS01_SAM | 2.14 | 1.49 | 2.68 | 1.71 | 2.72 |
| 2dyaB_BS01_ADP | 3.05 | 2.51 | 2.89 | 3.79 | 3.56 |
| 2ovdA_BS01_DAO | 2.39 | 2.41 | 1.18 | 1.67 | 1.87 |
| 3cpaA_BS01_III | 2.11 | 1.97 | 3.19 | 2.04 | 2.78 |
| 3stdA_BS01_MQ0 | 1.95 | 1.12 | 1.49 | 2.49 | 1.69 |

DockEM, ChemEM and EMERALD denotes the RMSD of using density maps with resolution range from 10 Å to 15 Å.
